# Influence of Herbicide Exposure and Ranavirus Infection on Growth and Survival of Juvenile Red-Eared Slider Turtles (*Trachemys scripta elegans*)

**DOI:** 10.3390/v13081440

**Published:** 2021-07-23

**Authors:** Rachel M. Goodman, Edward Davis Carter, Debra L. Miller

**Affiliations:** 1 Biology Department, Hampden-Sydney College, Hampden-Sydney, VA 23943, USA; 2 Center for Wildlife Health, University of Tennessee Institute of Agriculture, Knoxville, TN 37996, USA; ecarte27@utk.edu (E.D.C.); dmille42@utk.edu (D.L.M.); 3 Department of Biomedical and Diagnostic Sciences, College of Veterinary Medicine, University of Tennessee, Knoxville, TN 37996, USA

**Keywords:** atrazine, Roundup, Rodeo, glyphosate, reptile, iridovirus

## Abstract

Ranaviruses are an important wildlife pathogen of fish, amphibians, and reptiles. Previous studies have shown that susceptibility and severity of infection can vary with age, host species, virus strain, temperature, population density, and presence of environmental stressors. Experiments are limited with respect to interactions between this pathogen and environmental stressors in reptiles. In this study, we exposed hatchling red-eared slider turtles (*Trachemys scripta elegans*) to herbicide and ranavirus treatments to examine direct effects and interactions on growth, morbidity, and mortality. Turtles were assigned to one of three herbicide treatments or a control group. Turtles were exposed to atrazine, Roundup ProMax^®^, or Rodeo^®^ via water bath during the first 3 weeks of the experiment. After 1 week, turtles were exposed to either a control (cell culture medium) or ranavirus-infected cell lysate via injection into the pectoral muscles. Necropsies were performed upon death or upon euthanasia after 5 weeks. Tissues were collected for histopathology and detection of ranavirus DNA via quantitative PCR. Only 57.5% of turtles exposed to ranavirus tested positive for ranaviral DNA at the time of death. Turtles exposed to ranavirus died sooner and lost more mass and carapace length, but not plastron length, than did controls. Exposure to environmentally relevant concentrations of herbicides did not impact infection rate, morbidity, or mortality of hatchling turtles due to ranavirus exposure. We also found no direct effects of herbicide or interactions with ranavirus exposure on growth or survival time. Results of this study should be interpreted in the context of the modest ranavirus infection rate achieved, the general lack of growth, and the unplanned presence of an additional pathogen in our study.

## 1. Introduction

In the last two decades, biologists have become alarmed about potential impacts of iridoviruses, which infect and can cause mass mortality events in reptiles, amphibians, and fishes [1]. Iridoviruses are double-stranded DNA viruses that replicate in temperatures ranging from 12–32 °C and may survive several months outside of any host in aquatic environments [2]. Studies on iridovirus pathogenesis and disease ecology have demonstrated that susceptibility and severity of infection vary with age, host species, virus strain, temperature, population density, and presence of environmental stressors [3,4,5,6,7,8,9,10]. Among fish, iridovirus infections have been reported on several continents and can cause economic damage in commercial freshwater fisheries [9,11,12]. In amphibians, ranaviruses (family *Iridoviridae*, genus *Ranavirus*) have caused more die-offs in North America than the better-known fungus, *Batrachochytrium dendrobatidis* (*Bd*) [13,14]. The impact of ranaviruses on reptilian population dynamics is largely unknown, although several cases of morbidity and mortality in captive and natural populations have been attributed to this pathogen (reviewed in [12,15,16,17]).

Studies on ranavirus in turtles have included isolation of ranaviruses from natural populations and reports of deaths and declines in captive and wild species [12,18,19]; experiments have examined susceptibility and transmission of ranavirus between species and the importance of exposure dose and rearing temperature to host susceptibility and mortality [18,20,21,22,23,24]. While ranavirus outbreaks with high mortality may result in disconcerting headlines, limited surveys have also found ranavirus infection occurring at low prevalence in populations without apparent die-offs [25,26,27]. Ranavirus is now well documented on six continents [12], but significant questions remain regarding distribution and factors influencing morbidity and mortality in wild reptile populations. Experimental studies examining the effect of environmental stressors on ranavirus infection are especially lacking in reptiles. Thus far, temperature is the only environmental variable that has been experimentally investigated for impact on ranavirus susceptibility and survival in reptiles [28].

The current study examined potential interactions among a common environmental stressor, herbicides, and ranavirus exposure in juveniles of a largely aquatic turtle, *Trachemys scripta elegans*. This species has a wide geographic distribution in central and eastern US, occupies diverse habitats including bogs, ponds, lakes, and rivers, spends ample time in the water [29], and can be a reservoir for ranaviruses that infect more susceptible species, including those from other vertebrate classes (reviewed in [12,20]). Research on infectious diseases in chelonians is needed because over half of species in this taxon are listed as vulnerable, threatened, or endangered according to the IUCN [30].

Pesticides are a multibillion-dollar annual industry and are used more heavily in the Unites States than in many other countries; these products are found in over half of the country’s streams and many other surface waters [31,32,33]. Herbicides and insecticides enter aquatic systems via runoff from agricultural and urban uses and via direct application to water bodies [34]. In aquatic systems, these chemicals can have profound impacts on the biodiversity and productivity of and interactions among organisms [35]. Chemical pollutants can impact host–pathogen interactions by deactivating or killing the pathogen, or by directly and indirectly weakening the host immune response, thus increasing susceptibility to disease [36,37]. Stress hormones such as cortisol and corticosterone can become elevated in response to environmental pollutants and decrease host immune response, which increases susceptibility to infectious diseases [38,39]. Commonly used pesticides applied at sublethal doses can reduce immune response and resistance to parasites [40,41,42,43]. Pesticides have also been shown to negatively impact growth and survival, and they can change sexual characteristics in amphibians and reptiles [44,45]. Thus far, the interaction between pesticides and ranavirus infection have been studied in amphibians [46,47,48] but not in reptiles.

We exposed juvenile turtles to three commonly used herbicides that contain two different main active ingredients, glyphosate and atrazine, to determine whether these chemicals altered growth rates and susceptibility, morbidity, and mortality due to ranavirus exposure. Glyphosate is the most heavily used herbicide in the United States, due largely to the increasing dominance of crops that are genetically engineered to be resistant to this herbicide [49,50]. Surfactants, which help the herbicide to penetrate plant leaves, are common ingredients of these products, yet are generally not tested or listed on labels although they can be equally or more toxic than glyphosate [44,51,52]. Therefore, we examined glyphosate exposure via water bath containing two glyphosate formulations: Roundup ProMax^®^, which is formulated for terrestrial use and, therefore, contains surfactants, and Rodeo^®^, which is formulated for aquatic use without surfactants. Atrazine is the second most commonly used herbicide in the United States and is the most common pesticide found in US streams and groundwater [32,53]. Because of its impacts of growth, development, and reproduction in various species and persistent half-life of up to 19 months in water, this herbicide has been banned from use in the European Union since 2004 [36,54]. However, controversy exists surrounding the nature and magnitude of atrazine impacts in ecological communities [55,56,57].

Because of the aforementioned impacts of glyphosate and atrazine on reptilian growth, development, and survival, as well as the interaction of pesticides and ranavirus in studies conducted on amphibians, we expected similar effects in our experiment with *T. s. elegans*. Specifically, we predicted that exposure to herbicides would negatively impact growth and survival and increase morbidity and mortality due to ranavirus infection relative to controls not exposed to herbicide treatments.

## 2. Materials and Methods

We obtained 170 juvenile red-eared slider turtles from a commercial supplier (Reptile City; Dallas, TX, USA) in May of 2014. Ten turtles were immediately euthanized by injection of >130 mg/kg sodium pentobarbital injection into the supravertebral (subcarapacial) vein, followed by decapitation 1 h after injection. These turtles were necropsied to obtain the following tissues which genomic DNA was then extracted from: approximately 4 cm of mid- and distal intestine, the left kidney, and the left lobe of liver. Testing via quantitative polymerase chain reaction (qPCR; described below) for ranaviral DNA was performed to ensure that supplied animals were not infected with ranavirus. Upon confirmation of negative results for the subsample of 10 turtles, the remaining 160 turtles were placed individually in 5 L plastic containers containing 350 mL of filtered, dechlorinated tap water.

Laboratory temperatures were maintained between 19.5–28 °C with an average temperature of 24 °C. A natural photoperiod (approximately 6:00 a.m.–8:30 p.m. for May–June in Virginia) supplied by large windows in the room was supplemented by overhead fluorescent lights during 9:00 a.m.–6:00 p.m. Throughout the experiment, we fed turtles approximately 10% of their weight in Zoo Med natural aquatic turtle feed every other day. Turtle enclosures were placed on shelves in our laboratory and rotated weekly to avoid any bias due to position within the laboratory. Paper dividers were placed between each container and on the outside edge, to reduce possible stress.

After 3 days of acclimation to the laboratory conditions, turtles were assigned to one of eight experimental treatments (*N* = 20 for each) in a full factorial design with two factors: ranavirus exposure (control, ranavirus-exposed) and herbicide exposure (control, Roundup ProMax^®^, Rodeo^®^, atrazine). At the initiation of the experiment, we collected initial weights and measurements of each turtle (mass in g; width and plastron and carapace length in mm using digital calipers) and collected 3–4 mm from each tail tip using disposable sterile scalpel blades and sealed the wound with KwikStop Styptic Powder (containing benzocaine anesthetic; Miracle Care, Dayton, OH, USA). These tissue were collected to sample initial conditions in the event that any turtles not exposed to ranavirus during the experiment turned out to be ranavirus-positive at the conclusion. However, this scenario did not occur; thus, these tail tissues were not tested for ranavirus.

Herbicide exposure consisted of herbicide addition (or lack thereof, for controls) in 200 mL of water in housing containers during the first 3 weeks of the experiment. Herbicide-free dechlorinated water was used for all turtles during the remaining 2 weeks of the 5 week experiment. Levels of herbicide exposure were based on the higher end of but not maximal concentrations reported in the literature: 2000 μg a.e./L glyphosate for Roundup ProMax^®^ and Rodeo^®^ [58,59] (Monsanto, Creve Coeur, MO, USA) and 20 μg/L for atrazine (Hi-Yield Voluntary Purchasing Groups, Bonham, TX, USA) [53,60,61].

To constitute treatment water for glyphosate herbicides, 33.4 μL of Rodeo and 29.6 μL of Roundup ProMax^®^ were each added to 500 mL of water and mixed on a stir plate on high for 10 min. We then added each 500 mL stock solution to 7500 mL of water in a large bucket and stirred each by hand for 5 min. For the atrazine treatments, we added 20.1 μL of Hi-Yield Atrazine^®^ to 600 mL and mixed this on a stir plate on high for 10 min. An aliquot of 100 mL of this stock solution was then added to 7900 mL of water in a large bucket and stirred by hand for 5 min. In two different weeks, we took samples from the three final herbicide solutions and shipped them overnight on ice to the Mississippi State University Chemical Laboratory to verify target concentrations. Actual concentrations of water samples were 2048–2223 ppb and 1952–2292 ppb for glyphosate in Roundup Pro Max^®^ and Rodeo^®^, respectively, and 22–27 ppb for atrazine.

Ranavirus exposures were conducted 1 week after initiation of the experiment. Because water bath exposure to ranavirus can result in low infection rates (0–20% in three turtle species [62], we used injection of ranavirus as our exposure method to maximize the potential for infection [28,63,64,65]. Ranavirus exposure was executed at the start of the second week by injection of 100 μL of virus-infected cell lysate (6.3 × 10^4^ TCID_50_; FV3 ranavirus culture originally isolated from a box turtle isolate grown in fathead minnow cells (similar procedure described in [28]), with 50 μL injected into each pectoral muscle. Control turtles were each injected with 50 μL of Dulbecco’s modified Eagle medium (used for fathead minnow cell culture; Thermo Fisher Scientific, Waltham, MA, USA) in each pectoral muscle.

Each week, turtles were weighed, measured, and placed in cleaned, disinfected tubs with reconstitutions of herbicide treatments for the first 3 weeks or herbicide-free dechlorinated water for the last 2 weeks. After virus exposures, turtles were checked visually every 12 h for external signs of ranavirus infection: lethargy, respiratory stress, cutaneous erythema, and ocular/nasal discharge. During weekly measurements and water changes, turtles were examined while in hand for ranaviral disease signs. When turtles expired before the completion of the experiment, they were examined and sampled as described below following euthanasia done at the conclusion of the experiment. Turtles were considered deceased when unresponsive to stimulation of the limbs and corneas. We weighed and measured, took note of and photographed any external signs of disease, and then decapitated and necropsied the turtles. The color and condition of the liver was noted, and tissue samples were taken from the intestine, kidney, and liver, as previously described. Tissues were frozen at −80 °C until use in ranavirus testing. The remainder of the turtle was preserved in 10% buffered formalin for histopathological examination. The experiment was concluded after 5 weeks (4 weeks after virus exposures), when all remaining living turtles were euthanized, measured, and sampled as described above.

We extracted DNA from tissue samples with Qiagen DNeasy Blood and Tissue Kits (Qiagen, Hilden, Germany). We standardized the amount of genomic DNA used in each reaction with an Epoch spectrophotometer (Biotek, Winooski, VT, USA). We tested for presence of ranavirus DNA using quantitative polymerase chain reaction (qPCR) following the protocol of Picco et al. [64]. Each 25 μL PCR reaction contained: 7 μL volume of combined nuclease-free water and genomic DNA (volume specific to each individual for 50 ng DNA); 12.5 μL of TaqMan Universal PCR Master Mix (Applied Biosystems™, Foster City, CA, USA); 1.5 μL each of 10 μM primers: F 5′–ACA CCA CCG CCC AAA AGT AC–3′, R 5′–CCG TTC ATG ATG CGG ATA ATG–3′; 2.5 μL of 2.5 μM probe: 5′–/56-FAM/CCT CAT CGT /ZEN/TCT GGC CAT CAA CCA /3IABkFQ/–3′ (Integrated DNA Technologies, Coralville, IA, USA). All samples were tested in duplicate using Applied Biosystems™ StepOne Real-time PCR machine with two negative and two positive controls in each run (pure water and DNA extracted from cultured FV3 ranavirus). Samples with Ct values <30 for both runs were considered positive for ranavirus, according to standards established for this machine using known negative and positive controls from water, cultured ranavirus, and ranavirus-infected reptiles [25,26]. If Ct values from two samples for an individual were not both <30 or if one approximated 30, we ran two additional PCR reactions. Then, the consensus from three out of four total reactions was used.

We conducted histopathology on four turtles from each combination of herbicide and virus exposure (eight treatments, 32 turtles total). Four turtles with the lowest Ct values were selected from each of the ranavirus-exposed treatment groups, and we performed histological examinations of commonly infected tissues including the liver, spleen, pancreas, and gastrointestinal tract. All turtle tissues were processed at the University of Tennessee Veterinary Medical Center Diagnostic Laboratory where they were embedded in paraffin blocks, cut into 5 μm sections, mounted on glass slides, and stained with hematoxylin and eosin. We examined tissues under light microscopy for signs of ranaviral infection, as well as possible tissue lesions caused by herbicide exposure. All work in this study was approved by the Hampden-Sydney College Animal Care and Use Committee, under the protocol number 965 (approved 05 December 2014).

**Figure 1:**
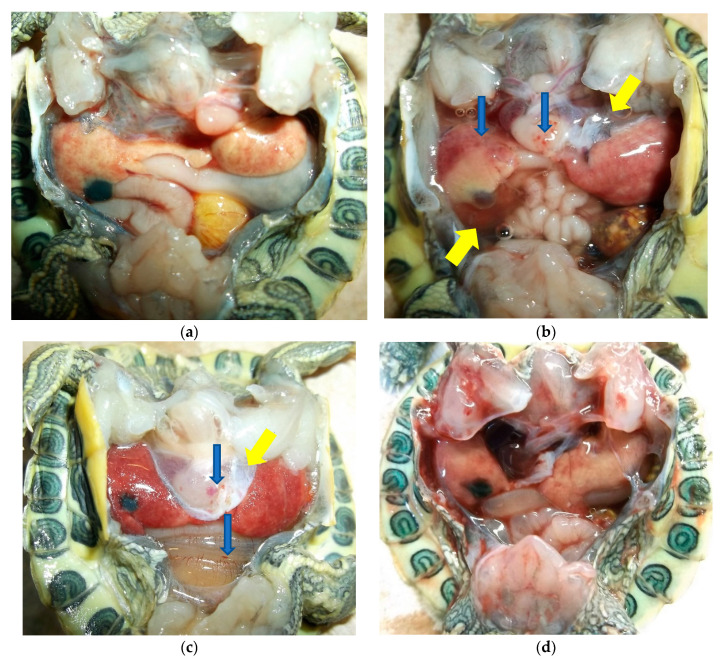
Representative images from necropsies of hatching turtles (*Trachemys scripta elegans*), showing liver discoloration due to ranavirus infection and herbicide exposure versus control conditions: (**a**) Turtle 41 (no herbicide, no ranavirus) control; (**b**) Turtle 78 (Rodeo, ranavirus-exposed) with petechial hemorrhaging on serosal surfaces (blue arrows) and edema fluid expanding soft tissue (yellow arrows); (**c**) Turtle 18 (no herbicide, ranavirus-exposed) with petechial hemorrhaging on serosal surfaces (blue arrows) and edema fluid expanding soft tissue (yellow arrows); (**d**) Turtle 13 (Rodeo, no ranavirus) control.

**Figure 2:**
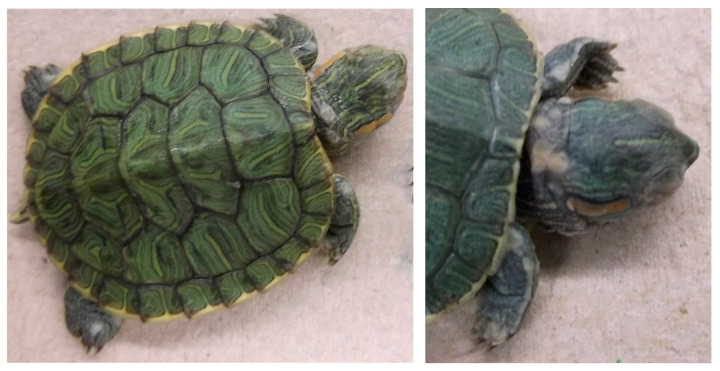
Representative images of white growths occurring on skin of hatching turtles (*Trachemys scripta elegans*), suspected as an unidentified fungal pathogen. Two different individuals are shown, with growths visible on the limbs and neck.

We used chi-squared tests of independence to compare ranavirus-positive and -negative PCR results among herbicide treatments and to compare incidence of external white growths (presumed fungus) and liver discoloration among herbicide treatments and ranavirus exposure treatments. To examine potential treatment effects of days survived and change in turtle mass, as well as plastron and carapace length, we used a weighted least squares regression model with herbicide and ranavirus exposure and an interaction effect as fixed factors, and time (day of measurement) as a fixed factor. We conducted our analyses in SPSS^®^ Statistics version 23 (SPSS Inc., Chicago, IL, USA) and created Figure 1 and Figure 2 in R version 4.0.0 (R Foundation for Statistical Computing, Vienna, Austria) [66].

## 3. Results

**Figure 3:**
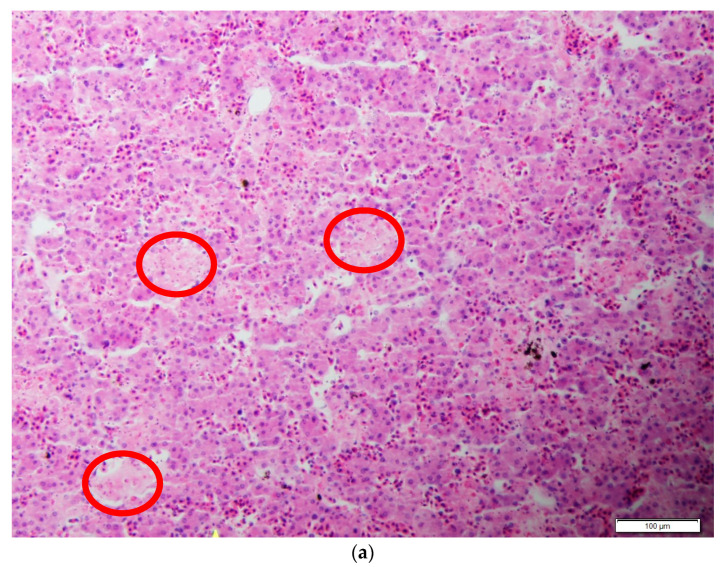

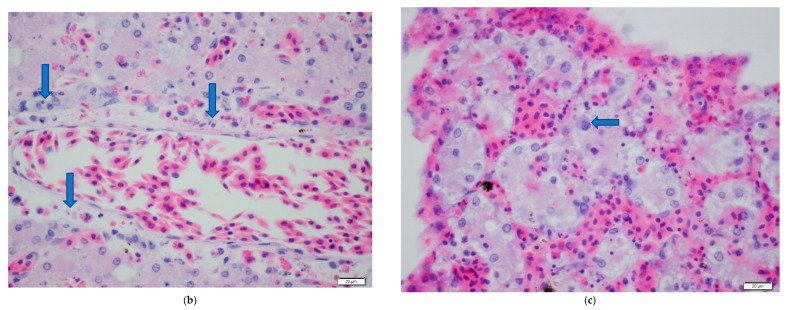

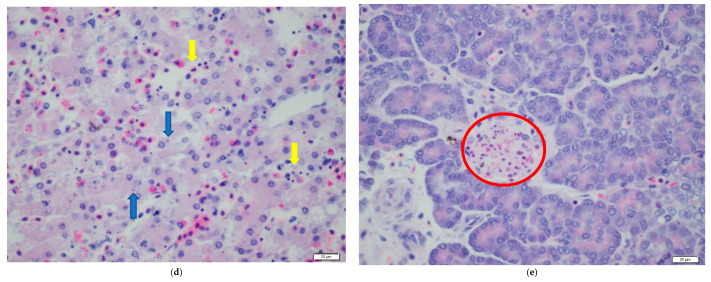

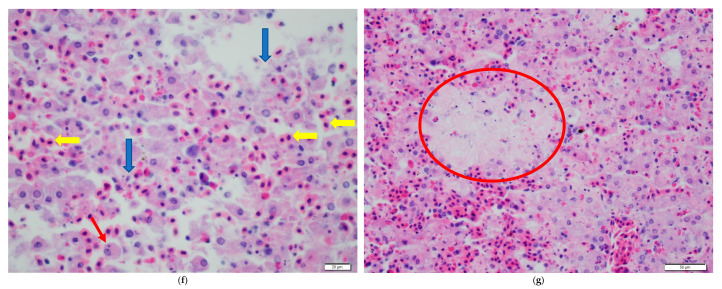
Representative tissues from hatching turtles (*Trachemys scripta elegans*) experimentally exposed to herbicides and ranavirus. Hematoxylin and eosin stain. Images are large and labels are on each for the purpose of manuscript review. (**a**) Turtle 40 (atrazine, ranavirus-exposed), liver showing areas of necrosis; (**b**) Turtle 48 (atrazine, ranavirus-exposed), liver showing necrosis of hematopoietic tissue and intracytoplasmic inclusions in granulocytes; (**c**) Turtle 62 (Rodeo, ranavirus-exposed), liver showing inclusion body consistent with ranavirus; (**d**) Turtle 66 (no herbicide, ranavirus-exposed), showing inclusions in hepatocytes (blue arrows) and hematopoietic necrosis (yellow arrows); (**e**) Turtle 76 (Roundup, ranavirus-exposed), showing hematopoietic necrosis in pancreas; (**f**) Turtle 94 (Rodeo, ranavirus-exposed), liver showing hematopoietic and hepatocellular necrosis (blue arrow), intracytoplasmic inclusion body (red arrow), and hemorrhage (yellow arrows); (**g**) Turtle 122 (no herbicide, ranavirus-exposed), liver showing hepatocellular necrosis.

Herbicide treatment did not affect incidence of ranavirus infection among turtles exposed to ranavirus (χ^2^ = 6.79, df = 3, *p* = 0.079), although there was a nonsignificant trend for higher infection rates in the herbicide control group relative to other groups infected with ranavirus (Table 1). Neither variable influenced liver color (tan or red), which was observed in 69 out of 160 turtles at the time of necropsy (χ^2^ = 1.13, df = 3, *p* = 0.770; Table 1; Figure 1). Neither herbicide treatments nor ranavirus exposure treatments affected the incidence of flocculent white growths (typically circular; 1–3 mm in diameter), which were observed in 65 out of 160 turtles at the time of necropsy (χ^2^ = 1.69, df = 3, *p* = 0.640; Table 1; Figure 2). These growths were not observed upon receipt of the shipment of turtles, but they were observed starting on the 10th day of the experiment and culminated in 1–23 growths per turtle, occurring on the skin but not the shells. We attempted to examine the white growths under microscopy but were unable to determine the causal agent. Histopathological changes in ranavirus-exposed turtles with the highest viral loads (lowest Ct values) included necrosis (Figure 3a,b,d,f,g) and hemorrhage in the liver (Figure 3f), hematopoietic necrosis in the pancreas (Figure 3e), and inclusion bodies consistent with ranavirus (Figure 3b–d,f).

**Figure 4:**
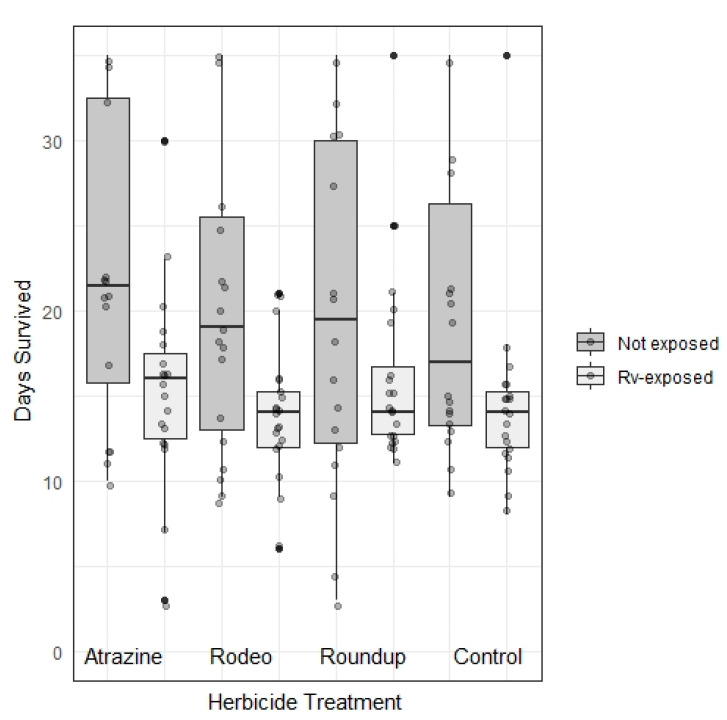
Days survived for hatchling turtles (*Trachemys scripta elegans*) exposed to herbicide and ranavirus treatments. Rv-exposed turtles were injected with 100 uL of ranavirus-infected cell lysate (FV3; 6.3 × 10^4^ TCID_50_) into the pectoral muscles after 1 week of the experiment; controls were injected similarly with cell culture medium. Turtles were exposed to herbicides via water bath during the first 3 weeks of the experiment. All remaining turtles were euthanized at the conclusion of the 5-week experiment.

**Figure 5:**
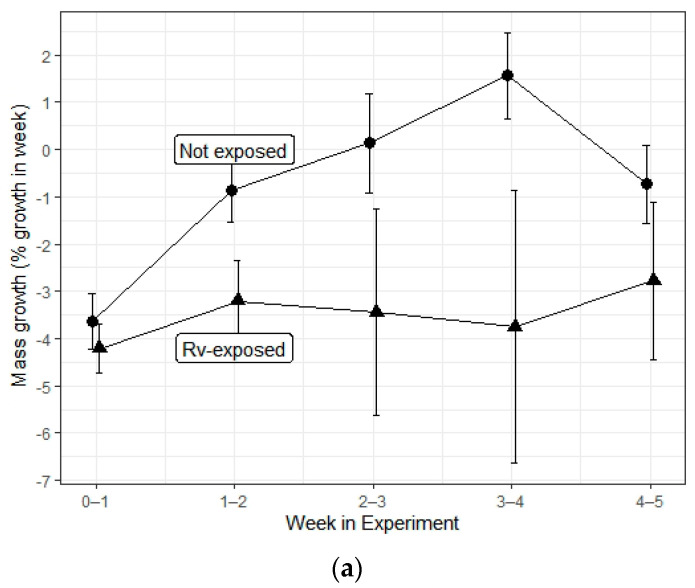

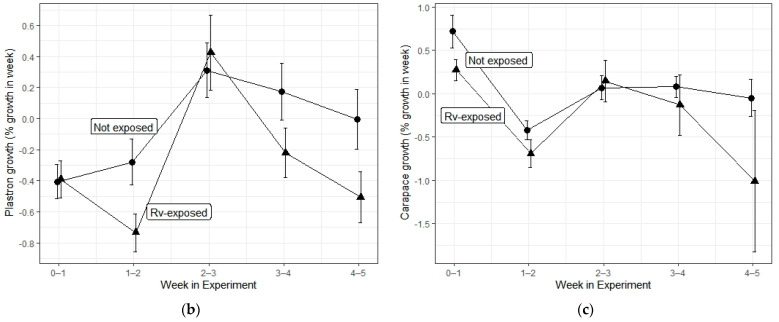
Response variables for hatchling turtles (*Trachemys scripta elegans*) in a 5-week experiment wherein they were exposed to ranavirus (Rv-exposed), or not, for controls. Rv-exposed turtles were injected with 100 uL of ranavirus-infected cell lysate (FV3; 6.3 × 10^4^ TCID_50_) into the pectoral muscles after 1 week of the experiment; controls were injected similarly with cell culture medium used for culturing the virus. Turtles were exposed to herbicides via water bath during the first 3 weeks of the experiment; all groups are combined here because these treatments had no impact on the response variables shown here on the *y*-axes: change in mass (**a**), plastron (**b**), and carapace (**c**). Points represent the mean (±1 SE) for Rv-exposed and control hatchlings for each week of experiment.

Ranavirus exposure decreased the number of days survived; however, there was no effect of herbicide treatment nor interaction effect between ranavirus and herbicide exposures (Table 2; Figure 4). The average starting sizes of hatchlings turtles were 7.26 g (SD = 0.97), 33.32 mm in carapace length (SD = 1.54), and 32.07 mm in plastron length (SD = 1.53). Ranavirus exposure negatively impacted change in mass and carapace length; however, there was no effect of herbicide treatment nor interaction effect (Table 2; Figure 5a,c). Plastron length was not impacted by either treatment or interaction between them (Table 2). Turtles lost a smaller percentage of body mass in control groups (mean = −1.6, SE = 0.4% per week) relative to ranavirus-exposed groups (mean = −3.3, SE = 0.5% per week). A similar nonsignificant trend was apparent for plastron length (Table 2; Figure 5b) in control groups (mean = −0.2, SE = 0.1% per week) relative to ranavirus-exposed groups (mean = −0.4, SE = 0.01% per week). Turtle carapaces grew slightly in control groups (mean = 0.2, SE = 0.1% per week) but shrank slightly in ranavirus-exposed groups (mean = −0.2, SE = 0.1% per week; Table 2; Figure 5c). Growth in all three measures varied significantly between weeks (Table 2), although no temporal trends were apparent (Figure 5).

## 4. Discussion

Our study demonstrated decreased survival time and greater loss of mass and carapace but not plastron length in juvenile turtles exposed to ranavirus as compared to controls. For mass and carapace length, the effect size for ranavirus exposure was minimal and represented less loss in turtles in control as compared to treatment groups. Although we fed turtles regularly, we suspect they were mostly subsisting on residual yolk stores, since we observed residual yolks in many turtles at the time of necropsy. Yolk remains were only visible at necropsy in turtles who died at or before 21 days into the experiment, with one exception. This timing is similar to a previous study of the same species [67] in which fasted hatchling used up all yolk remains by the end of 21 days post hatching. However, our turtles may have been up to 1 week older than indicated by the days of our experiment, since we purchased juveniles rather than hatching them from eggs as in [67]. We observed some feeding by juvenile turtles, although it was limited and variable (we did not quantify this behavior). Another study using *T. e. scripta* found little evidence of eating in the first 14 days post hatching [68].

Only 57.5% of turtles in our study that were exposed to ranavirus tested positive for ranaviral DNA at the time of death. Differences in infection rate from previously published studies may be attributable to characteristics of viral strains, inoculation dosage, housing conditions of turtles, or differences in tissues samples for PCR testing. Wirth et al. [23] also used intramuscular inoculation to infect turtle hatchlings (*Emydura macquarii krefftii*) with dosage ranging from 1 × 10^1.33^ to 1 × 10^5.33^ TCID (versus 6.3 × 10^4^ TCID_50_ in our study). Median survival time in that study was 22 days across all dose groups, and turtles inoculated with 1 × 10^4.33^ and 1 × 10^5.33^ TCID had a 100% infection rate as indicated by testing liver tissue for ranaviral DNA. Comparison of infection and mortality rates with that study may be complicated by the fact that they infected an Australian freshwater turtle with BIV (*Bohle iridovirus*) isolate. In another study using BIV, Ariel et al. [22] exposed hatchlings of two species of Australian tortoise (*Elseya latisternum* and *Emydura krefftii*) with intracelomic injections of 10^4.5^ TCID_50_. In the two species, respectively, three of five and eight of 12 exposed individuals died or were euthanized due to extreme lethargy at 10–29 days post inoculation. Among inoculated hatchlings, ranaviral DNA was only detected from two of five *E*. *latisternum* and four of 12 *E. krefftii.* Johnson et al. [21] injected 1 × 10^5^ TCID of Burmese star tortoise ranavirus (BSTRV) intramuscularly and found a 92% infection rate based on ranaviral DNA in kidney tissue (among eight hatchlings of *T. scripta elegans* and four hatchlings of *T. carolina carolina*). Allender et al. [24] injected hatching turtles with 5 × 10^5^ TCID_50_ of a FV3-like ranavirus isolated from an eastern box turtle (*Terrapene carolina carolina*); this represents a similar strain but higher dose relative to our study. In that study, which included our study subject *T. scripta elegans*, as well as three other species, ranavirus DNA was detected in all inoculated turtles, and deaths of all infected turtles occurred 6–16 days after inoculation. Allender et al. [28] injected 5 × 10^5^ TCID_50_ of a FV3-like virus intramuscularly into eight hatchings (*T. scripta elegans*) split into two rearing temperature treatments. All four turtles in the 28 °C treatment were euthanized due to severe clinical symptoms at 14–30 days post inoculation and contained ranaviral DNA in blood samples and oral and cloacal swabs. Only two of four hatchings in the 22 °C treatment developed clinical signs of infection and had detectable ranaviral DNA in blood samples and oral swabs. Comparing our infection rate of 57.5% with these studies, the most comparable studies were two studies by Allender et al. [24,28], which found infection rates of 100% and 75% using a similar ranaviral isolate, the same species, and hatchlings (10 days old or less) and adults. Our low rate of infection could be due to our inoculation dose of 6.3 × 10^4^ TCID_50_ which is roughly a magnitude lower than those studies or our average rearing temperature of 24 °C which is closer to the low-temperature treatment which resulted in a lower infection and mortality rate in [24,28]. Another difference from these studies is that we only sampled liver, kidney, and intestine tissues for PCR testing, whereas Allender et al. [24] sampled spleen, kidney, liver, and intestine tissues, and Allender et al. [28] sampled tongue, right forelimb skeletal muscle, liver, heart, lung, spleen, kidney, and ovary tissues.

In our experiment, we failed to detect any impact of environmentally relevant concentrations of atrazine, Roundup^®^, or Rodeo^®^ on infection rate, morbidity, and mortality of hatchling turtles due to ranavirus exposure. We also found no direct effects of herbicide or interactions with ranavirus exposure on growth or survival time. However, our results should be interpreted with caution because of the modest ranavirus infection rate achieved and the general lack of growth (which may reflect overall health) in our study subjects.

Some studies indicated no detrimental health impacts of atrazine on fish, amphibians, or reptiles when organisms were exposed to environmentally relevant concentrations (reviewed in [56]). However, studies also found that atrazine can have subclinical impacts on immune function, including that of our study species *T. scripta elegans* [36,69]. In an experiment with tiger salamanders (*Ambystoma tigrinum)*, atrazine caused higher ranavirus infection rates when *Ambystoma tigrinum* were challenged with *Ambystoma tigrinum* virus (ATV) at herbicide concentrations of 16 ug/L relative to 0, 1.6, and 160 ug/L [5]. For comparison, we used 20 ug/L for atrazine and found no impact on infection rate or other response variables despite using similar sample sizes per treatment group. Kerby and Storfer [48] exposed salamander larvae to atrazine (20 and 200 ug/L) and found that the herbicide slightly increased mortality rates in infected larvae but did not increase infection rates for ATV.

Although glyphosate toxicology has been studied in many wildlife species [70,71], little is known regarding interactions between glyphosate and ranavirus exposure. A recently published study found a higher percentage of mortality in juvenile hellbenders (*Cryptobranchus alleganiensis*) exposed to both ranavirus and Roundup in comparison to those only exposed to ranavirus; however, the differences in survival between these groups were not statistically significant [72]. Our study was limited to 4 weeks post exposure and measured only a few response variables: infection rate, survival, and growth. Although dramatic short-term impacts of glyphosate-based herbicides were not detected, this does not exclude the possibility of other changes that can have lasting impacts. For example, two environmentally realistic pulse exposures of Roundup WeatherMax^®^ altered mRNA levels of thyroid- and stress-related genes in wood frog tadpoles [73]. Some studies evaluating the impacts of glyphosate on reptile species observed increased DNA damage caused by exposure to the herbicide [74,75,76]. Investigations with *Caiman latirostris* found that Roundup^®^ reduced white blood cell counts, indicating that the herbicide can impact immune function in reptiles [77,78].

Impacts of glyphosate-based herbicides on amphibians may differ between chronic laboratory conditions or pulse exposure which better reflect environmental circumstances (reviewed in [73]). Chronic laboratory exposures, as in our study, have been criticized for representing a longer exposure period than in a natural setting; however, even under these circumstances, we failed to detect any negative impacts of Roundup ProMax^®^ or Rodeo^®^. Some studies of herbicide exposures use higher concentrations than those in our study (2000 μg a.e./L glyphosate). We conservatively chose levels that are commonly reported in the literature rather than record or maximal concentrations to mimic a more realistic level of environmental exposure.

The lack of growth and even a slight decrease in mass and length in our experiment are comparable to a study in which *T. e scripta* hatchlings were fasted in the first 3 weeks post hatching [67]. Few turtles remained (42 of 160) in the last 2 weeks of our study, when hatchling growth most likely would have occurred. Furthermore, many of those that were remaining showed evidence of skin infection caused by another pathogen (discussed below). Therefore, our power to detect potential differences in growth rates in later weeks of the experiments was limited. In a similar study by Allender et al. [24], mass of hatchling *T. scripta elegans* increased by 7% in only 16 days and did not vary between turtles exposed to ranavirus versus controls. Unfortunately, no growth data are available from the other studies of ranavirus-exposed hatchling turtles to compare with the current study [21,22,23].

Our study unintentionally included the presence of another pathogen, presumably a fungus, which caused small fuzzy white growths on the epidermis of hatchling turtles. Several turtles eventually had these growths on their eyelids, which appeared to cause irritation and swelling. These white growths were distributed evenly among treatments, and neither ranavirus nor herbicide exposures were associated with increased occurrence. Not providing hides or perches for the turtles to dry off during the study may have facilitated the growth this pathogen, which may have been acquired in our laboratory or prior to shipment of the turtles. We chose to house our turtles with constant exposure to a shallow aquatic environment to ensure consistent herbicide exposure between individuals. This stress may have also contributed to lack of growth in turtles in our study. We purchased juvenile turtles from a reptile supplier that describes its breeding facility as 12 turtle ponds containing approximately 1800 adult breeders of multiple species. The juveniles were housed in a small natural pond; thus, exposure to parasites, as well as stress experienced en route to our laboratory, may have affected the initial health of turtles in our experiment. We received the turtles in a box with soft loose cushion material and cloth bags containing 10 turtles per bag without any barriers or separation between them. The aforementioned potential stressors could have impacted the scope of the study; however, they were not expected to produce any systematic bias between treatments.

## 5. Conclusions

Ranaviruses are increasingly recognized as significant pathogens worldwide, but their influence on health and survival in reptiles is understudied [79]. Our study provides an investigation of interactions between ranaviruses and herbicides, but the importance of many other pollutants and environmental stressors remains to be examined. As expected, ranavirus exposure decreased survival time and reduced mass and plastron length in juvenile turtles. However, contrary to our expectations, we did not detect any direct effects of atrazine or two formulations of glyphosate on growth or survival or any interaction with ranavirus exposure in terms of susceptibility, morbidity, or mortality. We urge caution in interpretation of our results, because of the low growth rates and infection rates and the unplanned occurrence of another pathogen in our study system. In conclusion, we urge replication of ranavirus exposure studies in different study species and with inclusion of other environmental pollutants and stressors.

## Figures and Tables

**Table 1:** Incidence of response variables at time of death among hatchling turtles (*Trachemys scripta elegans*) exposed to herbicide and ranavirus treatments. Rv-exposed turtles were injected with 100 uL of ranavirus-infected cell lysate (FV3; 6.3 × 10^4^ TCID_50_) into the pectoral muscles after 1 week of the experiment; controls were injected similarly with cell culture medium. Turtles were exposed to herbicides via water bath during the first 3 weeks of the experiment. The criterion for ranavirus infection (incidence in table) was Ct values <30 for duplicate quantitative PCR wells using intestine, kidney, and liver tissue. Liver discoloration and external appearance of white growths were observed upon necropsy. Necropsies were performed upon death prior to the end of the experiment or upon euthanasia at the conclusion of the 5 week experiment.

Ranavirus Treatment	Herbicide Treatment	*N*	Incidence of Infection	Liver Discoloration	White Growths
Rv-exposed	Atrazine	20	12	12	9
Rv-exposed	Roundup	20	9	6	8
Rv-exposed	Rodeo	20	9	8	11
Rv-exposed	Control	20	16	10	12
Control	Atrazine	20	-	9	9
Control	Roundup	20	-	7	3
Control	Rodeo	20	-	10	6
Control	Control	20	-	7	7

**Table 2:** Results of GLM analysis with herbicide treatments, ranavirus exposure, and interaction effect between the two variables as fixed factors, and time (weeks 1–5) as a covariate. For herbicide treatments, hatchling turtles (*Trachemys scripta elegans*) were constantly exposed to four chemicals (atrazine, Roundup, Rodeo, and control) during the first 3 weeks of the experiment via water bath exposure. Ranavirus exposure consisted of injecting turtles with 100 uL of virus-infected cell lysate (FV3; 6.3 × 10^4^ TCID_50_) into the pectoral muscles after 1 week of the experiment; controls were injected similarly with cell culture medium.

Response Variables	Independent Variables	df	F	*p*
Days survived	Herbicide	3, 347	1.181	0.319
	Rv exposure	1, 347	31.366	<0.001 *
	Interaction	3, 347	0.235	0.872
Mass growth	Herbicide	3, 347	0.68	0.565
	Rv exposure	1, 347	7.933	0.005 *
	Interaction	3, 347	0.582	0.627
	Time	1, 347	17.886	<0.001 *
Carapace growth	Herbicide	3, 347	0.401	0.752
	Rv exposure	1, 347	6.861	0.009 *
	Interaction	3, 347	0.149	0.931
	Time	1, 347	13.539	<0.001 *
Plastron growth	Herbicide	3, 347	0.286	0.836
	Rv exposure	1, 347	2.761	0.098
	Interaction	3, 347	0.053	0.984
	Time	1, 347	8.529	0.004 *

*—Each asterisk indicates a significant relationship between the independent variable and response variable (*p* < 0.05).

## Data Availability

The data presented in this study are available online at: dx.doi.org/10.6084/m9.figshare.15032154.
